# Molecular Informatics Studies of the Iron-Dependent Regulator (ideR) Reveal Potential Novel Anti-*Mycobacterium ulcerans* Natural Product-Derived Compounds

**DOI:** 10.3390/molecules24122299

**Published:** 2019-06-21

**Authors:** Samuel K. Kwofie, Kweku S. Enninful, Jaleel A. Yussif, Lina A. Asante, Mavis Adjei, Kwabena Kan-Dapaah, Elvis K. Tiburu, Wilhelmina A. Mensah, Whelton A. Miller, Lydia Mosi, Michael D. Wilson

**Affiliations:** 1 Department of Biomedical Engineering, School of Engineering Sciences, College of Basic and Applied Sciences, University of Ghana, Legon P.O. Box LG 54, Accra, Ghana; kenninful@noguchi.ug.edu.gh (K.S.E.); jaleela96@yahoo.com (J.A.Y.); linaagyekumwaaa@gmail.com (L.A.A.); madjei008@st.ug.edu.gh (M.A.); kkan-dapaah@ug.edu.gh (K.K.-D.); etiburu@ug.edu.gh (E.K.T.); 2 West African Centre for Cell Biology of Infectious Pathogens, Department of Biochemistry, Cell and Molecular Biology, University of Ghana, Legon P.O. Box LG 54, Accra, Ghana; wamensah@gmail.com (W.A.M.); lmosi@ug.edu.gh (L.M.); 3 Department of Medicine, Loyola University Medical Center, Maywood, IL 60153, USA; wheltonm@seas.upenn.edu (W.A.M.III); Mwilson@noguchi.ug.edu.gh (M.D.W.); 4 Department of Parasitology, Noguchi Memorial Institute for Medical Research (NMIMR), College of Health Sciences (CHS), University of Ghana, Legon P.O. Box LG 54, Accra, Ghana; 5 Department of Chemical and Biomolecular Engineering, School of Engineering and Applied Science, University of Pennsylvania, Philadelphia, PA 19104, USA; 6 Department of Chemistry & Physics, College of Science and Technology, Lincoln University, Philadelphia, PA 19104, USA

**Keywords:** buruli ulcer, *Mycobacterium ulcerans*, iron dependent regulator (ideR), metal binding site, DNA–binding site, natural product compounds, molecular docking, molecular dynamics simulation

## Abstract

Buruli ulcer is a neglected tropical disease caused by the bacterium *Mycobacterium ulcerans.* Its virulence is attributed to the dermo-necrotic polyketide toxin mycolactone, whose synthesis is regressed when its iron acquisition system regulated by the iron-dependent regulator (ideR) is deactivated. Interfering with the activation mechanism of ideR to inhibit the toxin’s synthesis could serve as a possible cure for Buruli ulcer. The three-dimensional structure of the ideR for *Mycobacterium ulcerans* was generated using homology modeling. A library of 832 African natural products (AfroDB), as well as five known anti-mycobacterial compounds were docked against the metal binding site of the ideR. The area under the curve (AUC) values greater than 0.7 were obtained for the computed Receiver Operating Characteristics (ROC) curves, validating the docking protocol. The identified top hits were pharmacologically profiled using Absorption, Distribution, Metabolism, Elimination and Toxicity (ADMET) predictions and their binding mechanisms were characterized. Four compounds with ZINC IDs ZINC000018185774, ZINC000095485921, ZINC000014417338 and ZINC000005357841 emerged as leads with binding energies of −7.7 kcal/mol, −7.6 kcal/mol, −8.0 kcal/mol and −7.4 kcal/mol, respectively. Induced Fit Docking (IFD) was also performed to account for the protein’s flexibility upon ligand binding and to estimate the best plausible conformation of the complexes. Results obtained from the IFD were consistent with that of the molecular docking with the lead compounds forming interactions with known essential residues and some novel critical residues Thr14, Arg33 and Asp17. A hundred nanoseconds molecular dynamic simulations of the unbound ideR and its complexes with the respective lead compounds revealed changes in the ideR’s conformations induced by ZINC000018185774. Comparison of the lead compounds to reported potent inhibitors by docking them against the DNA-binding domain of the protein also showed the lead compounds to have very close binding affinities to those of the potent inhibitors. Interestingly, structurally similar compounds to ZINC000018185774 and ZINC000014417338, as well as analogues of ZINC000095485921, including quercetin are reported to possess anti-mycobacterial activity. Also, ZINC000005357841 was predicted to possess anti-inflammatory and anti-oxidative activities, which are relevant in Buruli ulcer and iron acquisition mechanisms, respectively. The leads are molecular templates which may serve as essential scaffolds for the design of future anti-*mycobacterium ulcerans* agents.

## 1. Introduction

Buruli ulcer is an infectious disease caused by *Mycobacterium ulcerans* [1]. It is a skin necrotizing disease that kills the cells of the skin and other soft tissues [2] and characterized by chronic ulceration of subcutaneous fat that leaves victims with unbearable deformity and disability when left untreated [3]. The pathogenesis of the disease starts as a painless nodule on the skin and may eventually grow into an extensive ulcer that can cover up to about 15% of an individual’s body. It is often referred to as the disease of the poor because most people stricken by the disease are inhabitants of poor rural communities with inadequate or no basic social amenities like potable water [4]. There are over 30 countries worldwide with reported cases of Buruli ulcer [5] and most of them are in Central and West Africa with few exceptions, including Australia. Cote d‘Ivoire, Ghana and Benin rank as the three countries with the highest prevalent rates [3]. About 1200 Buruli ulcer cases were reported in Ghana between 1993 and 1998 by a passive surveillance system established in the country. Between 2004 and 2014, reported cases exponentially increased to more than 9000 [6]. 

*M. ulcerans* is a slow growing bacterium doubling every 72 h [7] and like other slow-growing bacteria *Francisella tularensis* and *Borrelia burgdorferi*, it is classified as an environmental pathogen which implies that it survives in the external environment [8]. Its association with water and water bodies is well recognized and has generated hypotheses, such as bites of insects as the mode of transmission [2,9], which suggest mechanical transmission [10]. However, a recent study has demonstrated that acanthamoeba could be the host of the mycobacterium in the environment [11]. 

Despite the fact that the age or sex of the host have not been proven to be risk factors, statistics of recorded cases available suggest that the disease is more prevalent among women and children between the ages of 5 and 15 [3]. 

Virulence by *M. ulcerans* is attributed to the synthesis of a dermo-necrotic polyketide toxin called mycolactone [12]. The toxin is exported through the bacterial envelope and accumulates in an extracellular matrix [13]. It has also been shown to have immunosuppressive properties by inhibiting the phagocytic abilities of the phagocytic white blood cells and killing neutrophils dispatched to infected tissues [2,12]. Mycolactone also blocks exocytosis by blood platelets and mast cells, impairing wound healing processes [14]. 

Like all mycobacteria, *M. ulcerans* requires iron for growth [15]. Insufficient iron retards the growth of the bacterium and high intracellular level could cause irreparable oxidative damage [16]. The iron acquisition pathway of the mycobacterium ensures that an optimum amount of iron is taken in by the bacteria and this is regulated by the iron dependent regulator (ideR). Upon iron binding to ideR, it is activated and then binds to the iron boxes in the promoter regions of iron regulated genes, thereby deactivating iron acquisition (MbtB gene), activating iron storage (BfrB) and deactivating irtA (iron transport) and the reverse happens when iron levels are low. The binding of iron also induces structural changes in ideR, with the protein moving from an ‘open’ conformation in its inactive state to a ‘close’ conformation [15]. However, research has shown that a decrease in intracellular iron levels, which deactivates ideR reduces the synthesis of mycolactone [17]. This evidence led us to suggest that any molecule that targets the ideR to either prevent iron binding or induce conformational changes is potentially a drug. 

Natural products are chemical compounds that are produced by a living organism from nature which has the bioactivity capable to be used as drugs [18]. They represent an enormous reservoir of diverse sources of bioactive chemicals and is very essential to drug discovery [18].

This study employed computer-aided drug design techniques to screen for potential inhibitory compounds from an African natural product database (AfroDB). Also, undertake molecular modeling of the structure of ideR of *M. ulcerans*, as well as molecular dynamics simulations to identify compounds with the potential of inducing conformational changes in the ideR.

## 2. Results and Discussion

### 2.1. Three Dimensional (3D) Model Prediction and Validation 

**Figure 1:**
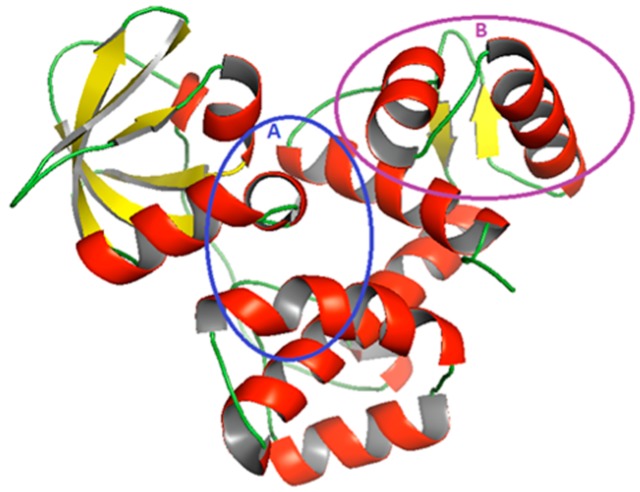
A cartoon representation of the 3D homology model of IdeR of *M. ulcerans*. Alpha helixes are colored in red, beta sheets in yellow and loops in green. Region ‘A’ circled in blue indicates the area of the metal binding pockets whilst region ‘B’, circled in violet shows the DNA–binding domain of the protein.

Using the amino acid sequence of the ideR for *M. ulcerans* (UniProt ID A0PT66) as a query to the Basic Local Alignment Search Tool (BLAST) [19], 23 hits were found from the Protein Data Bank (PDB) [20]. The best five of these 23 hits with a percentage sequence identity of 80% or more were selected (Table 1). Chain A of the crystal structure of the ideR from *M. tuberculosis* with PDB ID 1FX7 (1FX7_A) and 2.0 Å resolution was chosen as the template for modeling because it had the highest sequence coverage to the target and a reasonably good E-value of 8 × 10^−154^. Five models were predicted with Modeller version 9.17 using EasyModeller 4.0 [21] as an interface. The model with the lowest Discrete Optimized Potential Energy (DOPE) score was chosen as the best model. DOPE is an atomic distance-dependent statistical potential, which is calculated from a sample of native protein structures to assess the energies of generated protein models. Models with the lowest DOPE scores have the most stable minimized energy [22]. The list of generated models with their DOPE scores is shown in Table S1. The selected model 3 had the least DOPE score of −25,355.77734, and consists of 230 residues forming eleven alpha helices and seven beta sheets (Figure 1).

**Figure 2:**
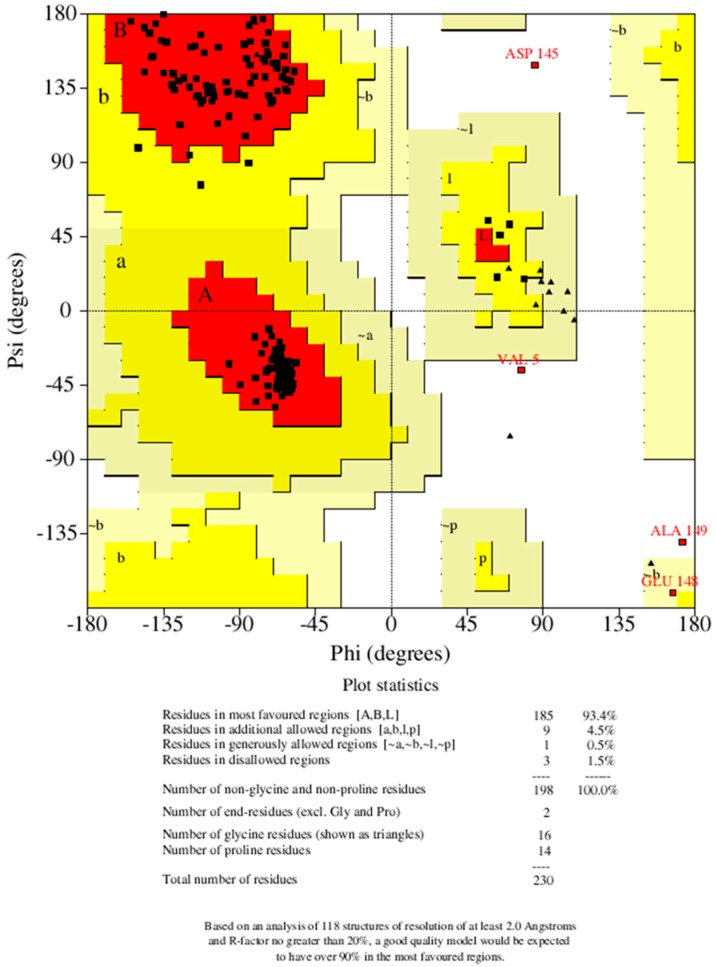
Ramachandran plot of ideR. This plot gives a general overview of the quality of the protein structure. The selected model is seen to have a reasonably good quality since most of its residues (>90%) fall within the favored region.

In validating the 3D model, the Ramachandran plot showed the percentage of residues in the allowed regions to be 5.0%, residues in the favored region to be 93.4% and residues in the outlier region to be 1.5% (Figure 2). A Ramachandran Z-score of 1.488 was obtained using WHATCHECK [23]. This indicates that our predicted model is of reasonably good quality since over 90% of residues were in the favored region [24]. The Z-score expresses how well the backbone conformations of all residues are corresponding to the known allowable areas in the Ramachandran plot [23]. A perfect Z-score is expected to be 1.0, and Z-scores above 4.0 and below −4.0 are very uncommon. Further verification with PROCHECK showed the resolution (normality) of the structure to be 1.5 Å which indicates a good resolution since most high-resolution X-ray structures have a resolution within 1.5 and 2.0 Å [24].

### 2.2. Binding Site Identification 

**Figure 3:**
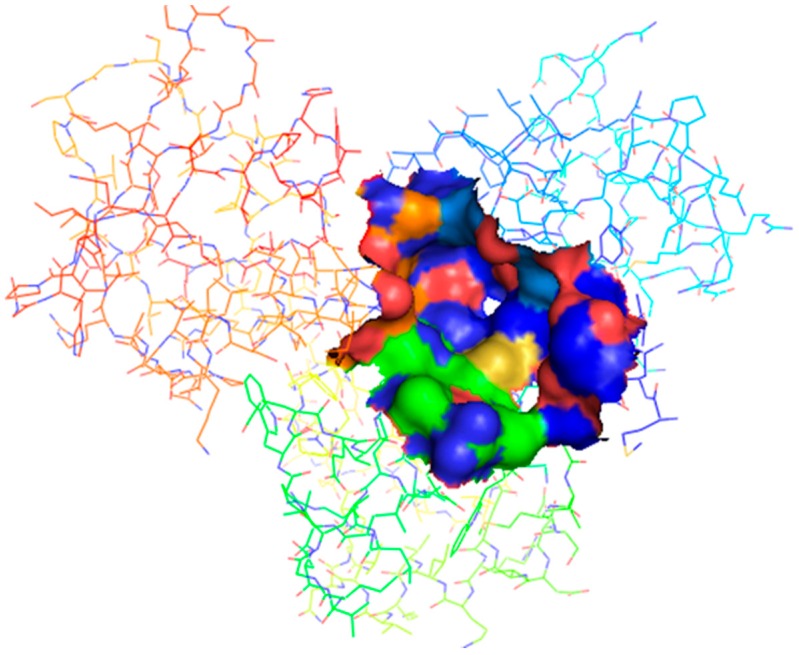
Metal Binding site 2 of the IdeR model. Binding pocket has been shown in surface representation. The image was generated with PyMOL molecular visualization tool.

The binding sites obtained from KVFinder, COACH and COFACTOR were compared with identified binding sites of co-crystallized structure, 1FX7_A, which was pre-processed and used as a template for the 3D model [23,24,25,26] (Table 2). The result obtained from COFACTOR provided a confidence score of 0.54, based on a range between 0 and 1, with values closer to 0 indicating a less reliable prediction and values closer to 1 indicating a more reliable prediction. From the value obtained, it can be concluded that the binding site predicted is reasonably reliable. The modeled structure had a binding site similarity (BS-score) of 1.9, a BS-score > 1 reflects a significant local match between the predicted and template binding site [27]. The COFACTOR server also provided a template modeling (TM) score of 0.999 for the modeled protein which showed that a good template was used. The TM score ranges between 0 and 1 with a better template having a value closer to one [28]. The result provided by COACH showed a confidence score of 0.01 which is less reliable. It also predicted the binding site residues as H219 and Y223. Two of the binding sites reported by Feese et al. [29] (binding sites 1 and 3) were similar to those predicted by COACH and COFACTOR. All the binding sites predicted by the KVFINDER matched four of those predicted in the template. Binding sites 1 and 2 were the most preferred binding sites since they both play essential roles in the activation of ideR as metal binding sites 1 and 2, respectively [13,14]. However, metal binding site 2 (Figure 3) proved to be the most suitable binding site because of its relatively large volume of 66.96 Å^3^ and surface area of 100.8 Å^2^, which may accommodate relatively large compounds.

### 2.3. Anti-Mycobacterial Lead Discovery

To the best of our knowledge, no known inhibitors have been screened against the ideR of *M. ulcerans*. However, five potent inhibitors comprising NSC303600 (IC_50_: 5.48 μg/mL), NSC12453 (IC_50_: 1 μg/mL), NSC65748 (IC_50_: 23.9 μg/mL), NSC201773 (IC_50_: 14 μg/mL) and NSC282699 (IC_50_: 24.3 μg/mL) have been identified against *M. tuberculosis* [30]. These compounds are from the National Cancer Institute (NCI) database and were screened against the DNA-binding site of the ideR of *M. tuberculosis*, a close homologue to the ideR of *M. ulcerans* [30]. A library composed of the five potent inhibitors and 832 compounds retrieved from AfroDB [31] were virtually screened against the metal binding site 2 of the modeled structure of the ideR, *M*. *ulcerans*.

**Figure 4:**
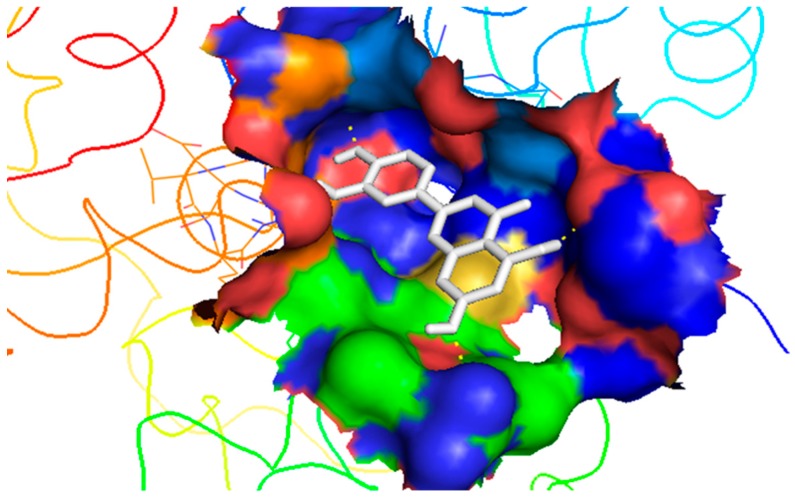
ZINC000018185774 firmly docked into metal binding site 2 of ideR. The binding site is shown as a solid surface whilst the ligand is shown in white stick model. The image was generated with PyMOL.

A total of 9272 different poses were generated from the compounds retrieved from AfroDB after the virtual screening. Hits were selected from the docking results by filtering with four criteria, which were drug likeness determined with Lipinski’s rule of five [32] and Veber’s rules [33], proper fit into the binding pocket, biomolecular interactions between ligands with binding site residues, and high binding affinity. Twenty compounds were shortlisted as top hits (Table 3). Figure 4 shows one of the selected hits firmly docked in the binding site. The biomolecular interactions between the five inhibitors and the binding site residues of the modeled ideR were also compared to those of the hits. 

The biomolecular interactions between the compounds and the metal binding site 2 of the modeled protein were generated with the processed_VinaResult.py script in the Autodock tools package, a python script useful for retrieving amino acid residues interacting with the docked ligand. We focused on residues located within the metal binding site of the ideR. The most common binding site residues with which the compounds interact with are Cys102 and Met10, an indication that these residues may perhaps play essential roles in the metal binding site. The five potent inhibitors, however, formed interactions with only His98, an amino acid residue of the metal binding site 1. All the Top 20 hits also formed interactions with His98, which indicates the amino acid could be very essential for ideR and may be involved in the activities of both metal binding sites.

### 2.4. Evaluation of Autodock Vina’s Performance

**Figure 5:**
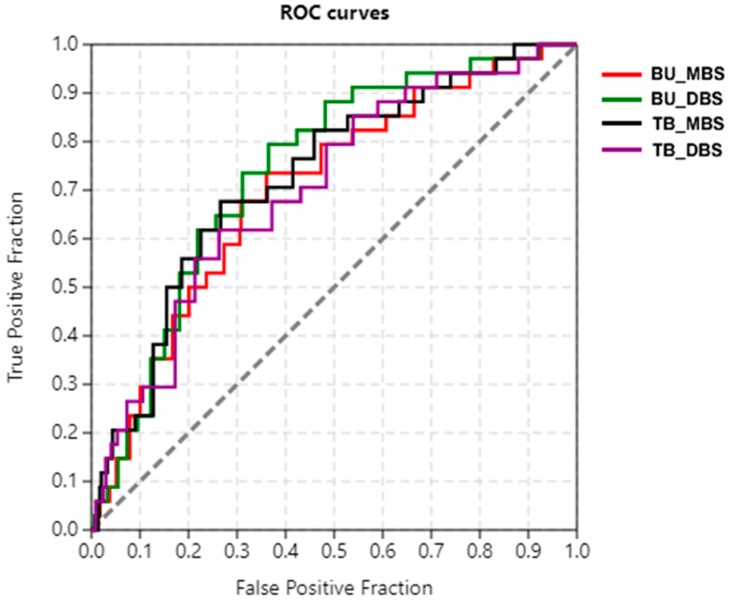
Receiver Operating Characteristic (ROC) curve for ideR of *M. ulcerans* and *M. tuberculosis*. Metal binding site 2 and DNA—binding site of ideR of *M. ulcerans* are shown in red and green, whilst that of *M. tuberculosis* are shown in black and violet, respectively. The curves were obtained after docking 34 actives and 1689 decoys against the respective binding sites.

The ability of Autodock Vina to accurately rank docked ligands was evaluated using different metrics comprising the area under the curve (AUC) of the Receiver Operating Characteristic (ROC) curve, Boltzmann-enhanced discrimination of ROC (BEDROC) and enrichment factors (EFs) [34]. The Receiver Operating Characteristic (ROC) curve is useful for measuring the ability of a docking or virtual screening software to distinguish between active and inactive compounds with respect to a receptor [32,33,34,35]. The performance of the docking is measured by calculating the area under the curve (AUC) value. The closer the AUC value is to 1, the better the ability of the docking to discriminate between active and inactive compounds. AUC value less than 0.5 is considered poor discrimination ability, 0.5 to 0.7 is moderate, 0.7 to 0.8 is acceptable, 0.8 to 0.9 is reasonably good and 0.9 to 1 is excellent [34,36]. The AUC values were obtained by screening 34 actives and 1689 decoys against metal binding site 2 and DNA–binding sites of the modeled ideR of *M*. *ulcerans* and that of the ideR of *M. tuberculosis*. The AUC of ROC (ROC_AUC) values obtained were above 0.70 for all the binding sites, which falls within the acceptable discrimination ability range (Table 4 and Figure 5). 

Since ROC evaluates the overall performance of the docking method to distinguish between actives and inactive (overall enrichment) [37,38,39], early enrichment was evaluated using the BEDROC and EFs (Table 4). BEDROC values obtained were between 0.1 and 0.2, whilst EF scores averaged around 2.7 for 1% to 20% of the screened compounds. BEDROC values obtained were higher than the expected value for random selection, which is 0.05 [40], BEDROC values greater than 0.5 are considered as the best performance for early enrichment. Even though, ROC_AUC values indicated that the overall performance of docking was successful in distinguishing between the entire library of actives and decoys, early recognition was suboptimal, as shown by EFs and BEDROC values (Table 4). Therefore, methods used for selecting hits considered the whole screened library and then predicted leads were consolidated with prediction of anti-mycobacterial activity, which was reinforced with structural similarity analysis of known anti-mycobacterials.

### 2.5. In Silico ADMET Studies

The in silico Absorption, Distribution, Metabolism, Elimination and Toxicity (ADMET) test profiles the pharmacokinetic, structural and physiochemical properties of small compounds [41]. The main aim of preclinical ADMET test was to eliminate weak drug candidates in the early stages of drug development, which helps in placing more emphasis on potentially better drug candidates [42]. In drug design, ligands are recognized by certain properties that distinguish drug-like and non-drug-like compounds [43]. Therefore, some of the properties that were considered were hydrogen bonds, solubility, bioavailability, molecular weight, permeability, toxicity, polar surface area, metabolism, and lipophilicity. The top 20 hits were evaluated using Free ADME-Tox Filtering Tool (FAF-Drug server) [41]. The compounds were scored as “accepted” (ligands with no structural alerts and satisfying the physicochemical filter), “intermediate” (ligands with low-risk structural alerts and few physiochemical properties below the threshold) and “rejected” (ligands that did not pass the physicochemical filter which include a high-risk structural alert and/or exceed the threshold of occurrence of low-risk structural alerts). The physicochemical filter used was Drug-likeness. Structural alerts are molecular substructures or reactive groups that are related to the carcinogenic and mutagenic properties of the chemicals and pose risks to compounds when used in drug discovery [44]. After the evaluation, two compounds were scored “accepted”, two were “intermediate” (Table 5) and 16 were “rejected”. Some of the structural alerts that were identified in most of the ‘rejected’ ligands included phenols, alpha (α) and beta (β)-unsaturated carboxylic acids, ketones, quinones and amides. The compounds that were considered “accepted” and “intermediate” were shortlisted as lead compounds (Table 5). Derek Nexus [41,42] provided an overall conclusion about the likelihood of lead compound toxicity by applying expert knowledge-based rules in toxicology (Table 6). Derek Nexus is a knowledge-based software that provides toxicity predictions in silico by identifying potentially toxic chemicals, which aids in the rejection of unsuitable drug candidates [45,46]. 

The pharmacological profiles of the lead compounds were then compared with that of five known Buruli ulcer drugs, namely Rifampicin, Streptomycin, Clarithromycin, Moxifloxacin, and Amikacin (Table 7). Lipinski’s violations, solubility and bioavailability of the compounds were computed. The leads violated none of the Lipinski’s rule of 5, whereas all the known drugs except moxifloxacin did not fully comply. The known drugs, however, had good solubility whilst the lead compounds had reduced solubility except ZINC000018185774. All the lead compounds, as well as rifampicin, moxifloxacin, and clarithromycin, had good bioavailability whilst the rest had low bioavailability. These results show some considerable difference between the properties of the known drugs and the lead compounds especially in the Lipinski’s rule of five. 

### 2.6. Molecular Dynamics (MD) Simulations

**Figure 6:**
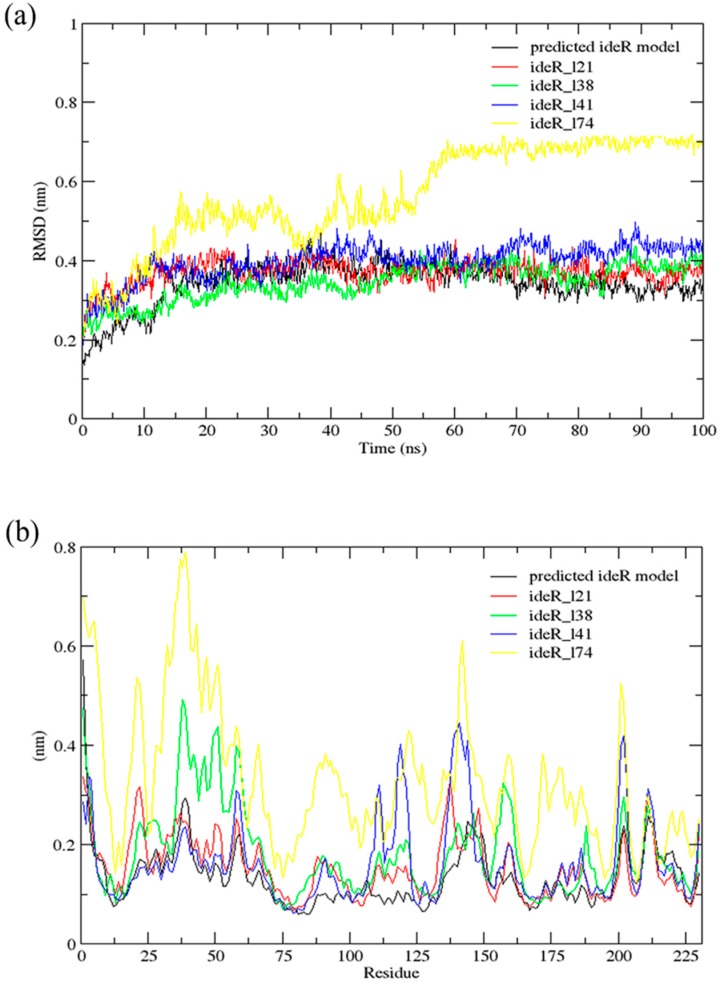
Root Mean Square–Deviation (RMSD) and –Fluctuation (RMSF) graphs of the respective complexes and generated a model of ideR; (**a**) represents the RMSD graph versus time and (**b**) shows the RMSF per residue graphs. Graphs are represented in different colours and indicated in the legend; black–predicted model of ideR, red–ider_l21 (ZINC000095485921-ideR complex), green–ider_l38 (ZINC000014417338-ideR complex), blue–ider_l41 (ZINC000005357841-ideR complex), and yellow–ider_l74 (ZINC000018185774-ideR complex).

A 100 ns MD simulation using GROMACS [47] was performed for the complexes of ideR and each lead compound and the results were compared to that of the unbound protein. This was done to investigate any influence on the structural conformation of the protein, due to the binding of the predicted lead compounds. Root Mean Square Deviation (RMSD) and RMS–Fluctuation (RMSF) graphs were generated after the simulations and the results of the complexes and the unbound ideR were compared (Figure 6). The RMSD graph accounts for the deviation of the atoms of the protein from the backbone of the protein and RMSF shows the movement of the protein residues during the simulation [48]. From the RMSD graph (Figure 6a), it was observed that most of the complexes experienced little fluctuations throughout the simulation with RMSDs close to that of the unbound protein. The RMSDs of ideR_l21, ideR_l38, ideR_l41 and unbound ideR structure fell between 0.3 nm and 0.4 nm from 20 ns, with ideR_l21 showing the closest RMSD fluctuations to that of the unbound protein. However, ideR_l74 endured very huge fluctuations in the first 60 ns of the simulation. The RMSD of ideR_l74 rose to 0.5 nm from 0–15 ns and again to 0.7 nm within the 55th–60th ns, where it remains stable till the end of the simulation. This indicates that the protein structure may have experienced conformational changes induced by the binding of ZINC000018185774. The RMSF graph (Figure 6b) affirms this observation, as huge fluctuations can be observed in the residues’ positions of the ideR_l74 complex. The fluctuations of the ideR_l74 peak the highest for residues within the DNA–binding domain of ideR (residues 1 to 75); the overall fluctuations with respect to that of the unbound ideR indicate high instability in the protein’s structure, due to its binding to ZINC000018185774. Some fluctuations were observed in the other complexes though not as high as that of ideR_l74. The complex of ideR_l38 showed some high fluctuations for residues 30–60 whilst ideR_l41 showed high fluctuations for residues between 100 and 150. On the other hand, ideR_l21 showed very high stability since it experienced minimal fluctuations as compared to the others and was close to that of the unbound ideR. Induced changes in the protein’s conformation by a ligand, particularly ZINC000018185774 can disrupt the protein’s iron acquisition system causing difficulty in the mycobacterium’s survival within its host. 

### 2.7. Induced Fit Docking

**Figure 7:**
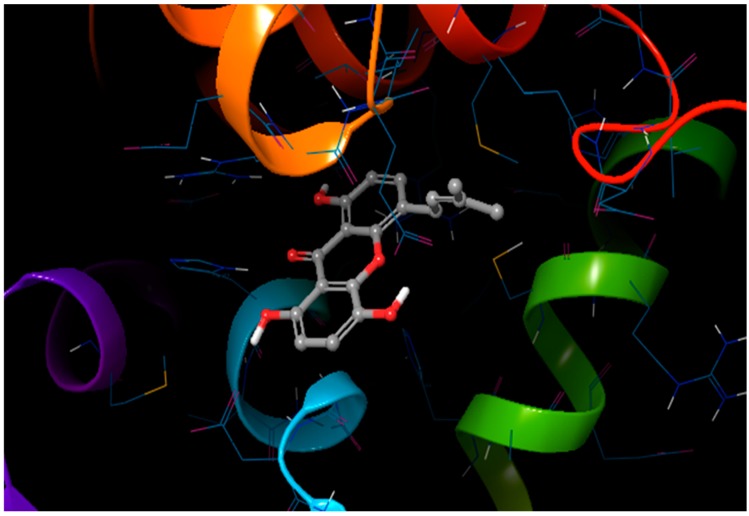
The induced fit pose of ZINC000095485921 (grey) in metal binding site 2 of the ideR of *M. ulcerans* model.

**Figure 8:**
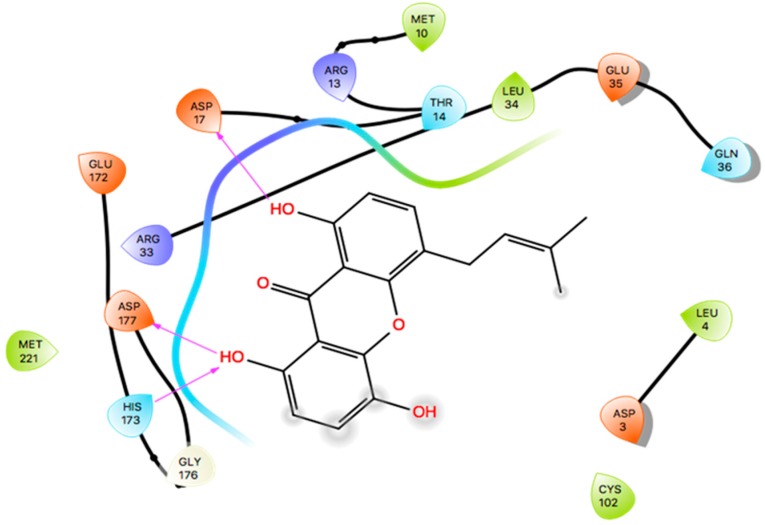
Two–dimensional interaction map of ZINC000095485921–ideR complex obtained after IFD. Hydrogen bonds are shown in violet lines with arrow heads.

**Figure 9:**
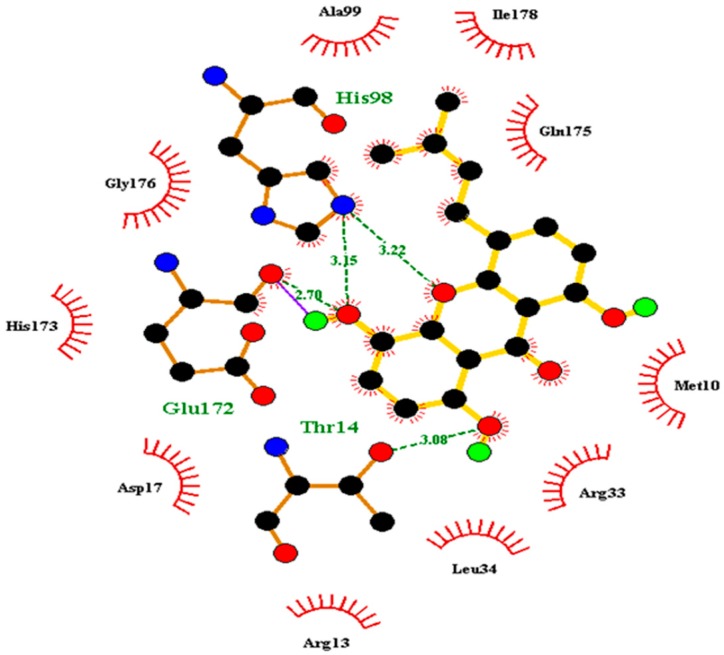
Ligplot of ZINC000095485921 showing the ligands interactions pre–IFD. Ligand is shown in yellow and hydrogen bonds are shown in green broken lines. Refer to Figure S1 for the ligplots of the other lead compounds.

Docking has become a widely accepted and standard method in computational drug discovery. It is, however, limited by difficulties like modeling flexibility of the protein upon ligand binding [49,50]. As such, Induced Fit Docking (IFD) was employed for the lead compounds complexed with ideR of M. *ulcerans*, since IFD models protein flexibility upon ligand binding [51,52]. IFD scores and Glide scores were obtained for the complexes. The IFD scores estimate the best plausible conformation of the ligand complex and the Glide scores gives a measure of the binding affinity between a ligand and a receptor [53,54]. A more negative value in both the IFD and Glide scores represent a more plausible conformation and better binding of the protein–ligand complex respectively. ZINC000095485921, ZINC000018185774, ZINC000014417338 and ZINC000005357841 complexes obtained Glide score of −7.60 kcal/mol, −7.00 kcal/mol, −6.75 kcal/mol and −5.82 kcal/mol, as well as IFD scores of −471.96 kcal/mol, −471.27 kcal/mol, −471.05 kcal/mol and −464.79 kcal/mol, respectively. The Lead compounds formed interactions with residues, such as Met10, Glu172, Cys102, and His98, which have already been predicted as essential residues of the metal binding site after the virtual screening. They also formed interactions with Thr14, Arg33 and Asp17. These residues form hydrogen bond interactions with the lead compounds at metal binding site 2 (Table 8), providing insights into other novel residues which can be further exploited. The binding pose and interaction map of ZINC000095485921 are shown in Figure 7 and Figure 8, respectively, whilst those of ZINC000018185774, ZINC000014417338 and ZINC000005357841 are shown in Figures S3 and S4. The ligplot of ZINC000095485921 showing the interactions of the ligand in the Metal binding site 2 of the modeled ideR before IFD is also shown in Figure 9.

## 3. Screening of Lead Compounds and Known Inhibitors against the DNA-Binding Site

Selected lead compounds comprising ZINC000018185774, ZINC000095485921, ZINC000014417338 and ZINC000005357841 were screened against the DNA–binding site of the modeled ideR alongside the five known potent inhibitors (Table 8). This screening was done to investigate the activity of the chosen leads against the DNA–binding site of the modeled ideR in comparison to the potent inhibitors discovered for the *M*. *tuberculosis* ideR’s DNA–binding site. This was possible since both proteins are close homologues and showed high conservation at the DNA–binding domain. Binding energies obtained for the lead compounds ranged from −5.7 to −5.9 kcal/mol, which is very close to those of the five potent inhibitors (−5.5 to −6.0 kcal/mol) (Table 8). The hydrogen bond interactions between the compounds and the protein were also analysed using LigPlot^+^ [55] to investigate common interacting residues the lead compounds might share with the five potent inhibitors. It was observed that the lead compounds and the potent inhibitors shared common hydrogen bond interactions with Arg47, Arg27, Thr44 and Thr7. Ser37, Pro39, and Gln43, are essential residues within the DNA–binding site necessary for binding to DNA [30]. Two of the potent inhibitors, namely NSC12453 and NSC65748 formed hydrogen bond interactions with Gln43 and Ser37, respectively. None of the lead compounds formed hydrogen bond interactions with any of the three essential residues; however, ZINC000014417338 and ZINC000095485921 formed hydrogen bond interactions with Ser42, which has been shown to be a novel critical residue which could be exploited for discovery of inhibitors against ideR [30]. 

Induced Fit Docking was also performed for the binding of the lead compounds to the DNA–binding site to account for protein flexibility. ZINC000018185774, ZINC000014417338, ZINC000095485921 and ZINC000005357841 complexes obtained Glide score of −5.27 kcal/mol, −5.04 kcal/mol, −4.79 kcal/mol and −4.99 kcal/mol with IFD scores of −468.50 kcal/mol, −467.32 kcal/mol, −466.87 kcal/mol and −461.93 kcal/mol, respectively. The compounds formed interactions with common residues, such as Ala28, Arg60, and Ser42.

## 4. Exploring the Anti-Mycobacterial Activity of the Predicted Leads

Due to the limited financial resources for Buruli ulcer drug discovery, repurposing of antimycobacterials by screening against *M. ulcerans* is gaining attention [56,57]. A library of compounds from the tuberculosis lead generation and optimization programs was screened in a whole-cell assay against *M. ulcerans*, where five compounds were discovered to be potent inhibitors with high activity (IC_90_ ≤ 1 μM) [56]. Therefore, exploring the plethora of antitubercular structures to unravel potential anti-*M. ulcerans* scaffolds was adopted. Analogues, derivatives or structurally similar compounds to the leads were investigated for possible anti-mycobacterial related activities.

ZINC000018185774, also popularly known as luteolin, has been shown to exhibit anti-mycobacterial activity against *M. tuberculosis* via fractionations from crude samples of *Annona sylvatic* and *Ficus chlamydocarpa* with MIC values of 236.8 μg/mL and 78.12 μg/mL, respectively [58,59]. However, there was no report of the compound being tested against *M. ulcerans.* A structural similarity search performed in Drugbank revealed luteolin to be highly structurally similar to a flavonol compound, quercetin with a similarity score of 0.884. An analogue of quercetin, quercetin–3–*O*–β–d–glucoside has been reported to inhibit glutamine synthetase enzyme in *M. tuberculosis* (IC_50_ = 0.048 μM) [60]. Quercetin–3–*O*–β–d–glucoside (ZINC4096845) was casually docked against *M. ulcerans* of ideR and a high binding energy of −8.2 kcal/mol was obtained. The quercetin–3–*O*–β–d–glucoside docked firmly within the active site pocket of the *M. ulcerans’* ideR. We, therefore, suggest that both quercetin–3–*O*–β–d–glucoside and luteolin could be investigated as potential novel anti-Buruli ulcer leads.

ZINC000014417338, also popularly known as Alpinumisoflavone, has been reported to exhibit antibacterial and anti-mycobacterial activity with MIC value of 19.53 μg/mL against *M. smegmatis* [59]. Therefore, it is plausible to explore repurposing ZINC000014417338 as an anti-mycobacterial ulcerans. However, similarity search via Drugbank did not yield any structurally similar compound which exhibits anti-mycobacterial activity. This may be due to the stringent similarity threshold adopted for the query. Similarly, similarity searches for ZINC000095485921 (1,4,8-trihydroxy-5-(3-methylbut-2-enyl)xanthen-9-one) and ZINC000005357841 ((6-methoxybenzo [1,3]dioxol-5-yl)BLAHone) yielded no structure which has shown anti-mycobacterial activity. Even though, we could not find any report describing the anti-mycobacterial activity of ZINC000095485921, some novel 1,2,3-triazolyl xanthenones were reported to have shown good to excellent antimicrobial and anti-tubercular activity with MIC values from 3.12–6.25 μg/mL [61]. It is worth exploring ZINC000095485921 as a potential anti-mycobacterial lead, since it is also a xanthenone analogue. 

Due to the fact that no similar compounds or analogues were found for ZINC000005357841, the possible biological activity of the lead compound was predicted with Prediction of Activity Spectra for Substances (PASS) [62,63] and their Probable activity (Pa), as well as Probable inactivity (Pi) values were obtained. Among the results retrieved from PASS, the ones most relevant to anti-buruli ulcer activity and iron acquisition mechanisms were anti-inflammatory (Pa = 0.458, Pi = 0.07) and anti-oxidative (Pa = 0.305, Pi = 0.022) activities. The Pa values were greater than those of Pi obtained for both aforementioned biological activities, pointing out the need to further explore the pharmacological activity of ZINC000005357841 [62].

The results from the structural similarity searches and the enriched information obtained about the leads warrant experimental evaluation of their anti-Buruli ulcer activity. This study complements current efforts geared towards unravelling the mechanism of actions of potential Buruli ulcer drugs. Summary of lead compounds is shown in Table 9.

## 5. Materials and Methods

### 5.1. Homology Modeling of Mycobacterium ulcerans ideR Structure

The ideR of *M. ulcerans* protein had no experimentally solved structure in any of the protein databases, including the Protein Data Bank (PDB). Therefore, the 3D structure was generated using in silico homology modeling. The amino acid sequence of *M. ulcerans* (strain Agy99) with UniProt ID A0PT66 was used to acquire homologues as templates for modeling via the Basic Local Alignment Search Tool (BLAST) [19]. A suitable template with a good E-value and high sequence identity was selected from among the retrieved homologues as a template for modeling the protein target. The structure was modeled using Modeller version 9.17 embedded in EasyModeller 4.0 [21].

### 5.2. Structure Validation

The quality of the generated model was assessed using a Ramachandran plot and further validated with WHATCHECK and PROCHECK [21,22].

### 5.3. Binding Site (Pocket) Identification

After the quality of the model has been assessed to be reasonably accurate, putative binding sites were identified using KVFINDER, COFACTOR and COACH [23,24,25]. Predicted binding sites were also compared with the binding sites of the co-crystallised template [29] since proteins with similar folds are normally found to have similar binding sites.

### 5.4. Virtual Screening

Two stages of virtual screening were carried out for lead discovery. In the first stage, a library composed of 832 compounds retrieved from AfroDb [31] and five potent inhibitors of ideR of *M. tuberculosis* recently discovered [30], retrieved from the database of the National Cancer Institute (NCI), (Bethesda, MD, USA) were screened against the predicted binding site of the ideR. The compounds were energy minimized with OpenBabel in PyRx using the universal force field (uff) and conjugate gradients as the optimization algorithm with a total number of steps of 200. Virtual screening against the ideR model was done using AutoDock Vina embedded in PyRx version 0.8 [64] using grid box size of 24.3, 27.7, and 20.9 Å; as well as center dimensions of 4.5, 47.2, and −2.8 Å in the X, Y and Z coordinate axes, respectively. The second stage of virtual screening involved a library of identified potential leads and the five potent inhibitors. They were screened against the DNA–binding site of the modeled ideR with a grid box size of 25.0, 25.0, and 25.0 Å, as well as center dimensions of 13.11, 64.95, and −3.09 Å. An exhaustiveness of 8 was used for both screenings.

### 5.5. Validation of Docking Protocol

To validate the docking protocol used, a receiver operating characteristic (ROC) curve was generated by screening 34 actives [30] and their respective decoys against the Metal binding site 2 and DNA–binding sites of the modeled ideR of the *M. ulcerans* and the ideR of the *M. tuberculosis* (PDB ID: 1FX7) (Supplementary file S1). The actives are composed of the five potent inhibitors, as well as 12 other compounds and analogs of some of the highly performing compounds screened against the ideR of *M. tuberculosis* [30]. The decoys were generated with the Directory of Useful Decoys–Enhanced (DUD-E) [65]. A total of 1689 decoys were used together with the 34 actives for the screening. The docking results were used to generate the ROC curves and AUC values utilizing ROCKER [66] and screen explorer [67]. BEDROC values with an alpha of 20.0 and EFs at 1%, 10% and 20% were also evaluated.

### 5.6. In Silico ADMET Studies

FAF-Drug [41] and DEREK NEXUS version 2.1 [41,42] were used for ADMET analysis and ADME/tox elimination. The ligands were uploaded as Simplified Molecular Input Line Entry System (SMILES) or Structure Data File (SDF) and were scored as “accepted”, “intermediate” and “rejected”. The physicochemical filter used was Drug-likeness. DEREK NEXUS was used to further evaluate the toxicity profiles of the “accepted” and “intermediate” ligands obtained from FAF-Drug. The pharmacological profiles of the chosen leads were also compared to that of five known drugs.

### 5.7. Molecular Dynamic Simulations

GROningen MAchine for Chemical Simulation (GROMACS) version 5.1.1 [47] was used to perform the molecular dynamics simulations using the GROMOS96 43A1 force field. The ligands’ topology was, however, generated using PRODRG [68] since its topology could not be built in GROMACS. In running the simulation, the complex was first solvated in a 1 nm dodecahedron water box. The system was neutralized by adding 10 positive ions to balance the net charge of the complexes. The complex was then relaxed through energy minimization to remove any steric clashes or bad geometry. Thereafter, the system was equilibrated to the required temperature (300 K) and density (1020 kg/m^3^). After the system was equilibrated and set in the desired temperature and density, a 100 ns production run was then performed, and the results of the simulation were analysed using Xmgrace version 5.1.25. The unbound protein was also subjected to molecular dynamics using the same simulation parameters as those of the complexes with the OPLS force field.

### 5.8. Induced Fit Docking

Induced Fit Docking (IFD) of the lead compounds was done using the Schrödinger software suite and the GlideScores and IFD scores were generated for all the plausible poses. This was done to understand the flexibility of the modeled ideR since most docking programs dock a flexible ligand to rigid receptors [49]. The lead compounds were docked against the metal binding site 2 and the DNA–binding domain respectively.

### 5.9. Lead Structural Similarity Searches and Antimycobacterial Association

The popular names of predicted leads were retrieved from the ZINC database to investigate whether the compounds have been reported elsewhere to have shown anti-mycobacterial activity. This was undertaken to find reports of a possible relationship between the leads and mycobacteria. Also, SMILES files of the leads were used to retrieve structurally similar compounds present in the Drugbank [69]. The search was done with a similarity threshold of 0.7 and molecular size ranged between 100 Da and 500 Da. This was done to identify compounds with relatively high structural similarity to the queried leads, which may have known anti-mycobacterial activity and will further support the possibility of the predicted leads to exhibiting anti-mycobacterial activity. For further investigation of ZINC000005357841 since no report or analogue with anti-mycobacterial activity was found for it, ZINC000005357841 SMILES files were uploaded to the Prediction of Activity Spectra for Substances (PASS) online tool [63] to predict its biological activity.

## 6. Conclusions

African natural compounds ZINC000018185774, ZINC000095485921, ZINC000014417338 and ZINC000005357841 were identified as potential novel leads against the modeled structure of ideR of *M. ulcerans* by docking, which was validated with an AUC value of the ROC curve above 0.7. Five potent inhibitors of ideR of *M*. *tuberculosis* had similar binding energies as the leads when screened against the DNA–binding domain of the ideR of *M. ulcerans*. Novel critical residues of metal binding site 2 comprising Thr14, Arg33 and Asp17 were predicted. A hundred nanoseconds molecular dynamics simulations showed conformational changes in the ideR-ZINC000018185774 complex with implications in iron acquisition. Interestingly, quercetin, which is structurally similar to ZINC000018185774 was previously shown to exhibit antimycobacterial activity. Similarly, ZINC000014417338 (Alpinumisoflavone) was reported to exhibit anti-mycobacterial activity, whilst analogues of ZINC000095485921 have shown antimicrobial and antitubercular activity. ZINC000005357841 was predicted to possess anti-oxidant and anti-inflammatory activities. Since this work is largely computational, experimental confirmation of the anti-Buruli ulcer activity of the leads is critical. Furthermore, the scaffolds of the leads could be used for designing novel inhibitors.

## Figures and Tables

**Table 1:** Homologues of *Mycobacterium ulcerans’* Iron-dependent Regulator (ideR). These are best 5 of 23 hits with percentage identity from 80% and above obtained from Basic Local Alignment Search Tool (BLAST).

Hits Description	Query Cover	E Value	Identity	PDB ID
Chain A, Crystal Structure of ideR from *Mycobacterium tuberculosis*	100%	8 × 10^−154^	92%	1FX7_A
Chain A, Crystal Structure of a two-domain ideR-DNA complex crystal form I	64%	1 × 10^−96^	91%	2ISZ_A
Chain A, ideR from *M. tuberculosis*	60%	2 × 10^−95^	96%	1B1B_A
Chain A, Crystal Structure of the Nickel—activated two-domain Iron—dependent Regulator	64%	6 × 10^−95^	91%	2ISY_A
Chain A, Diphtheria Tox Repressor (C102d Mutant) complexed with Nickel and Dtxr consensus binding sequence	52%	1 × 10^−62^	80%	1F5T_A

**Table 2:** Summary of predicted binding sites of the model and reported binding sites of the template (1FX7, ideR from *M. tuberculosis*) and those predicted for the model via COACH and COFACTOR.

**BINDING SITE PREDICTION**	**RESIDUES AT THE BINDING SITE**
COFACTOR	His79, Glu 83, His 98, Glu172, Gln175
COACH	His219, His223
**REPORTED BINDING SITES OF THE TEMPLATE (1FX7) [29]**	
1 (Metal binding site 1)	His79, Glu83, His98, Glu172, Gln175
2 (Metal binding site 2)	Met10, Cys102, Glu105, His106
3	His219, His223
4	His212

**Table 3:** Table of Top 20 hits and five potent inhibitors with their binding energies and interacting metal binding site-2 residues. Residues were determined using the processed_VinaResult.py script in the Autodock tools package.

LIGAND ID	BINDING ENERGY	RESIDUES LIGAND INTERACTS WITH
NSC12453	−7.5	His98
NSC201773	−7.5	His98
NSC282699	−7.5	His98
NSC303600	−7	His98
NSC65748	−7	His98
ZINC000005357841	−7.4	Cys102, His98, Met10
ZINC000013327497	−7.1	Met10, Cys102, His98
ZINC000013481884	−7.3	Glu172, His98, Cys102
ZINC000014417338	−8	Glu172, His98
ZINC000014811038	−7.7	Glu172, His98, Cys102
ZINC000014819573	−7.4	His98, Cys102, Met10
ZINC000018185774	−7.7	His98, Cys102, Met10
ZINC000033831303	−7.4	Met10, Cys102, His98
ZINC000095485893	−7.2	Met10, His98, Cys102
ZINC000095485918	−6.9	Glu172, Cys102, His98
ZINC000095485921	−7.6	Met10, His98, Glu172
ZINC000095486065	−7.5	Met10, His98, Cys102, Glu172
ZINC000095486093	−7.1	Glu172, Cys102, His98
ZINC000095486151	−7.3	Cys102, Met10, His98
ZINC000095486157	−7	His98, Met10, Cys102
ZINC000095486193	−7.2	Glu172, His98, Cys102
ZINC000095486235	−8.3	His98, Cys102
ZINC000095486265	−7.8	Met10, His98, Cys102
ZINC000095486301	−7.6	His98, Met10, Cys102
ZINC000095486336	−8.4	Glu172, His98

**Table 4:** Respective values of ROC_AUC, BEDROC and Enrichment Factor for the metal binding site 2 and DNA- binding sites of ideR for *M. ulcerans* (BU_MBS and BU_DBS) and that of *M. tuberculosis* (TB_MBS and TB_DBS).

	ROC_AUC	BEDROC (alpha = 20.0)	Enrichment Factor		
			1%	10%	20%
**BU_MBS**	0.702	0.137	2.979	2.355	2.208
**BU_DBS**	0.743	0.143	2.979	2.355	2.650
**TB_MBS**	0.727	0.174	0	2.355	2.797
**TB_DBS**	0.703	0.175	5.95	2.650	2.355

**Table 5:** Free ADME-Tox Filtering results showing ligands that passed the physicochemical filtering rules with no structural alerts.

Ligands	Status
ZINC000005357841	Accepted
ZINC000014417338	Accepted
ZINC000018185774	Intermediate
ZINC000095485921	Intermediate

**Table 6:** Results obtained from Derek Nexus Software. The reasoning of outcome is indicated based on the confidence level of the predicted toxicological endpoint. The most common end point among the compounds was Skin sensitization which had a plausible outcome indicating a low confidence level.

Ligands.	End Point	Species	Reasoning Outcome	Negative Outcome	Strongest Ec3 Prediction
**ZINC000005357841**	Hepatotoxicity	Mammal	Plausible	-	-
	Mutagenicity In Vitro	Bacterium	-	Inactive	-
	Carcinogenicity	Mammal	Plausible	-	-
	Skin Sensitization	Mammal	Plausible	-	2.9% Moderate Sensitizer
**ZINC000018185774**	Teratogenicity	Mammal	Equivocal	-	-
	Mutagenicity In Vitro	Bacterium	-	Inactive	
	Skin Sensitization	Mammal	Plausible	-	0.15% Strong Sensitizer
**ZINC000095485921**	Photoallergenicity	Mammal	Plausible	-	-
	Teratogenicity	Mammal	Equivocal	-	-
	Mutagenicity In Vitro	Bacterium	-	Inactive	
	Skin Sensitization	Mammal	Plausible	-	0.15% Strong Sensitizer
**ZINC000014417338**	Mutagenicity In Vitro	Bacterium	-	Inactive	
	Skin Sensitization	Mammal	Plausible	-	0.16% Strong Sensitizer

**Table 7:** Pharmacological profiles of the lead compounds and five known drugs for Buruli ulcer. The known drugs are Rifampicin, Streptomycin, Clarithromycin, Moxifloxacin, and Amikacin.

Ligand ID	Lipinski’s Violation	Solubility (mg/L)	Solubility Forecast Index	Oral Bioavailability (Veber)
ZINC000014417338	0	3279.99	Reduced Solubility	Good
ZINC000005357841	0	4526.38	Reduced Solubility	Good
ZINC000018185774	0	8434.39	Good Solubility	Good
ZINC000095485921	0	2928.86	Reduced Solubility	Good
Moxifloxacin	0	30151.64	Good Solubility	Good
Amikacin	3	5077659.17	Good Solubility	Low
Streptomycin	3	3508974.65	Good Solubility	Low
Clarithromycin	2	1491.87	Good Solubility	Good
Rifampicin	4	246.01	Good Solubility	Good

**Table 8:** Binding energies and hydrogen bond interactions of selected lead compounds and the five potent inhibitors screened against metal binding site 2 and the DNA–binding site respectively. A hydrogen bond interaction was generated with LigPlot^+^ software.

Ligand ID	Metal Binding Site 2		DNA-Binding Site	
	Binding Energy (KCAL/MOL)	Hydrogen Bonds	Binding Energy (KCAL/MOL)	Hydrogen Bonds
NSC12453	−7.5	Gly176, Arg13, His98	−5.9	Gln43, Arg47, Thr7
NSC201773	−7.5	Gly176, His173, His98	−6	Arg27
NSC282699	−7.5	-	−5.9	Arg47, Thr44
NSC303600	−7	Thr14, Arg13, Arg33	−5.9	Thr8, Thr7, Asn2
NSC65748	−7	Arg33, Asp17, His173, Arg13	−5.5	Ser37, Thr40, Gln36, Glu35
ZINC000014417338	−8	Arg33, Asp17, His98	−5.9	Ser42
ZINC000018185774	−7.7	Asp3, Arg103, Arg33, Asp17, Glu172	−5.8	Arg47, Thr44
ZINC000095485921	−7.6	Thr14, His98, Glu172	−5.7	Arg60, Ser42, Arg27, Ala28
ZINC000005357841	−7.4	His98	−5.9	Thr7, Asn2, Thr44

**Table 9:** List of predicted lead compounds, their common names and two–dimensional structures obtained from Zinc database.

Ligand ID	Common Names	Two-Dimensional Structure
ZINC000014417338	Alpinumisoflavone; 5-hydroxy-7-(4-hydroxyphenyl)-2,2-dimethylpyrano[3,2-g]chromen-6-one	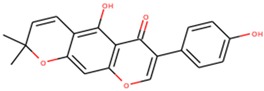
ZINC000005357841	(6-methoxybenzo[1,3]dioxol-5-yl)BLAHone	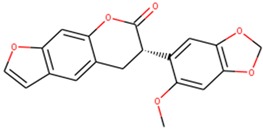
ZINC000018185774	Luteolin; 2-(3,4-Dihydroxy-phenyl)-5,7-dihydroxy-chromen-4-one	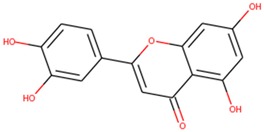
ZINC000095485921	1,4,8-trihydroxy-5-(3-methylbut-2-enyl)xanthen-9-one	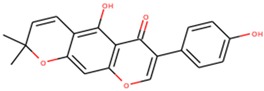

## Supplementary Materials

The following are available online at https://www.mdpi.com/1420-3049/24/12/2299/s1, Figure S1: Ligplots showing the residue interactions of the lead compounds docked in the metal binding site 2 of the modeled ideR for *M. ulcerans*. Figure S2: Induced fit docking poses of the ligands docked in the metal binding site 2 of the modeled ideR for *M. ulcerans*. Figure S3: Interaction maps (I–map) of lead compounds docked in the metal binding site 2 of the modeled ideR for *M. ulcerans* obtained after IFD. Table S1: Summary of successfully generated models with DOPE scores. Supplementary File S1: List of actives and decoys used for validation of docking method and screening results.
