# Supplementary material for: Molecular Informatics Studies of the Iron-Dependent Regulator (ideR) Reveal Potential Novel Anti-Mycobacterium ulcerans Natural Product-Derived Compounds
Source: Molecules. 2019 Jun 21;24(12):2299. doi: 10.3390/molecules24122299 (PMC6631925; doi:10.3390/molecules24122299)
Supplement: Supplementary file 1 [file molecules-24-02299-s001.zip › Supplementary_files_submitted_revsion_20_05_2019/Supplementary file_reviewed_FINAL_SKK.docx]

Table S1. Summary of successfully produced models with DOPE scores.

| Models | DOPE score |
| --- | --- |
| Model 1 | -25163.21680 |
| Model 2 | -25322.59375 |
| Model 3 | -25355.77734 |
| Model 4 | -25124.73828 |
| Model 5 | -25100.93164 |


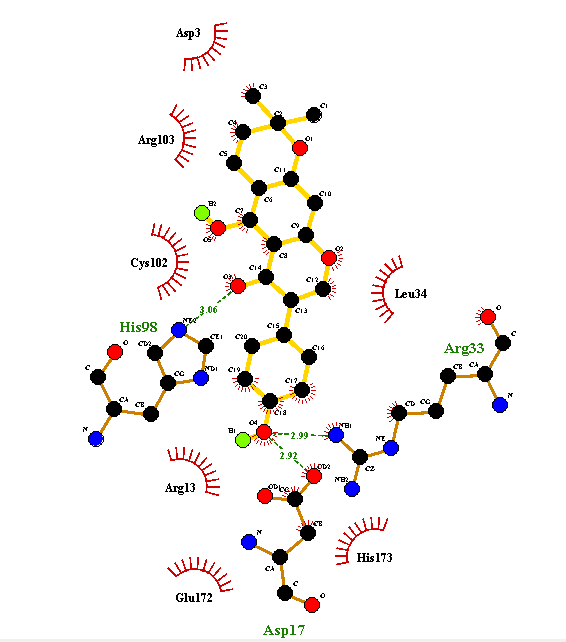

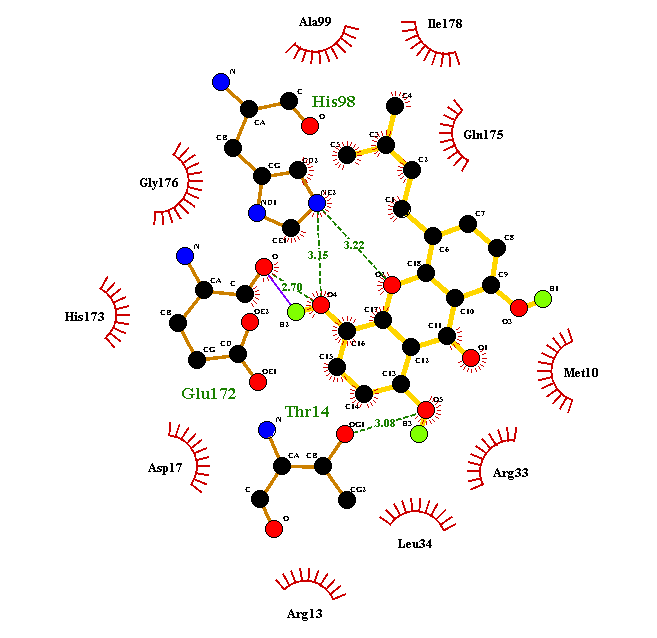
A B


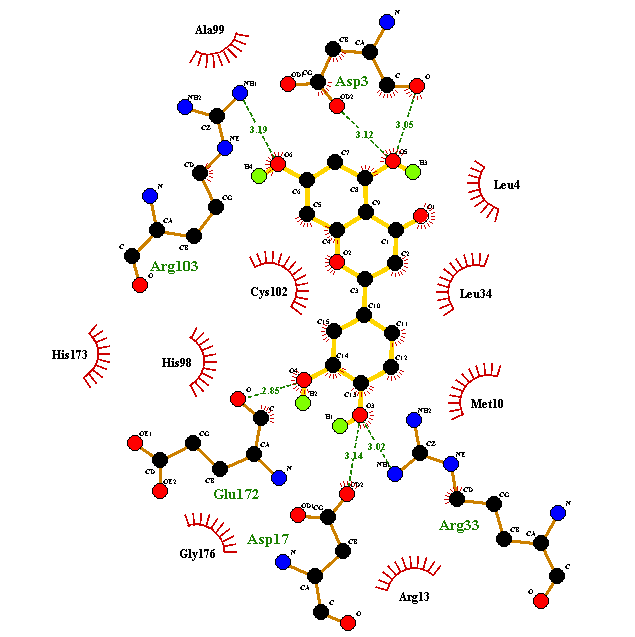

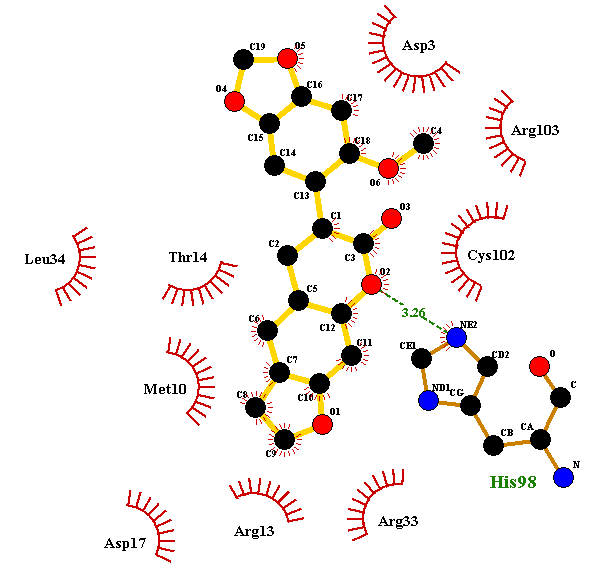
C D

Figure S1: Ligplots showing the residue interactions of the lead compounds docked in the metal binding site 2 of the modeled ideR for M. *ulcerans.* The ligplots of (A) ZINC000095485921; (B) ZINC000014417338; (C) ZINC000005357841; and (D) ZINC000018185774. Hydrogen bonds are shown as green broken lines and compound structures are represented with yellow colouring.


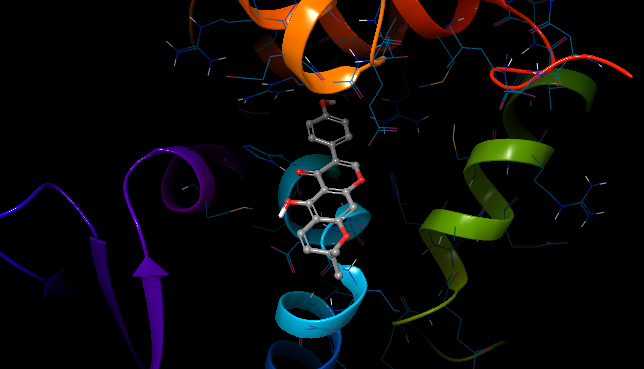

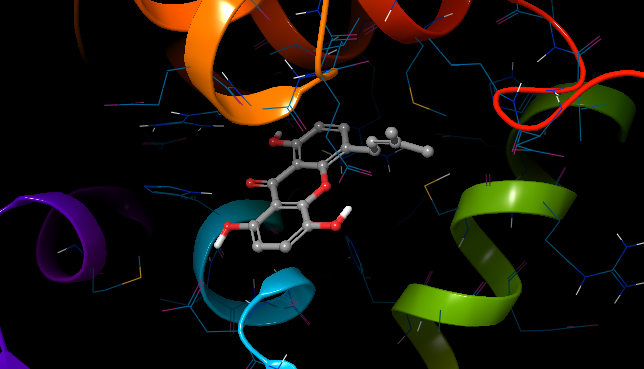
A B


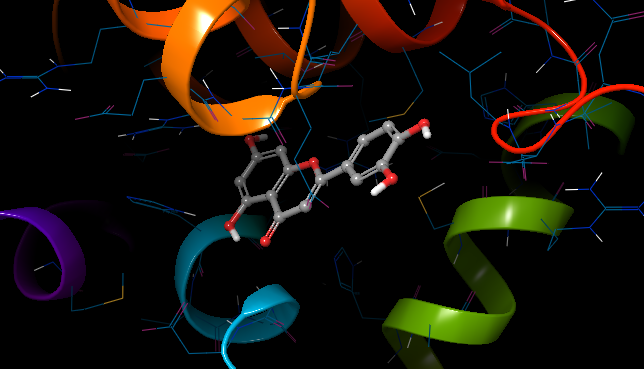

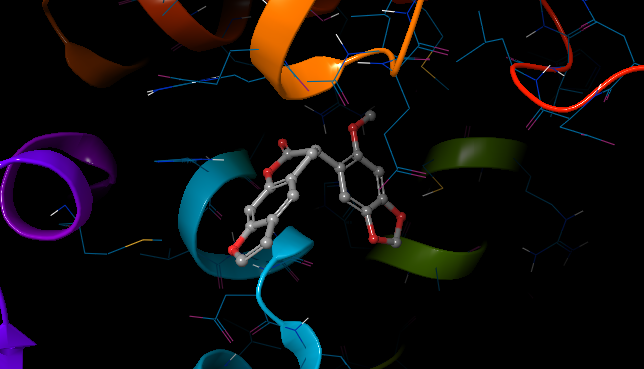
C D

Figure S3: Induced Fit Docking (IFD) poses of the ligand complexes of the lead compounds docked in the metal binding site 2 of the modeled ideR for M. *ulcerans.* The IFD poses of (A) ZINC000095485921; (B) ZINC000014417338; (C) ZINC000005357841; and (D) ZINC000018185774. Ligands are represented in gray.


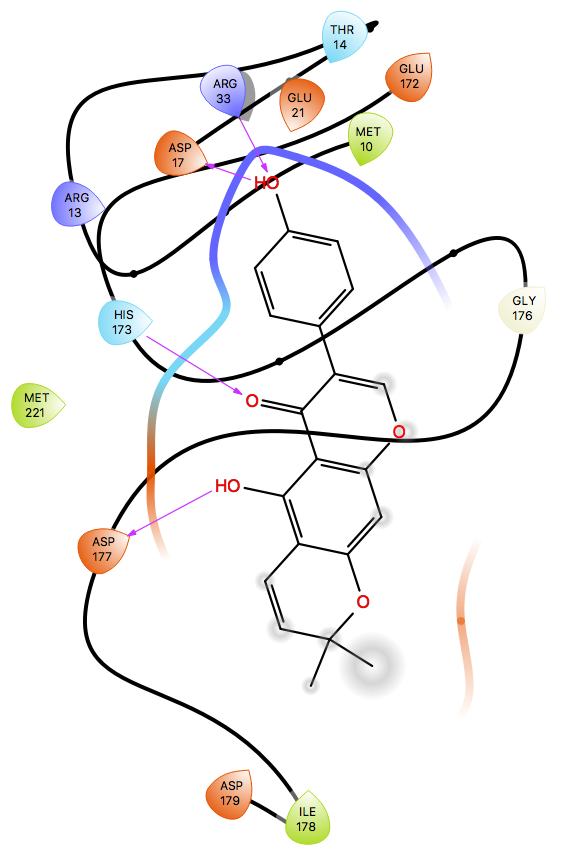

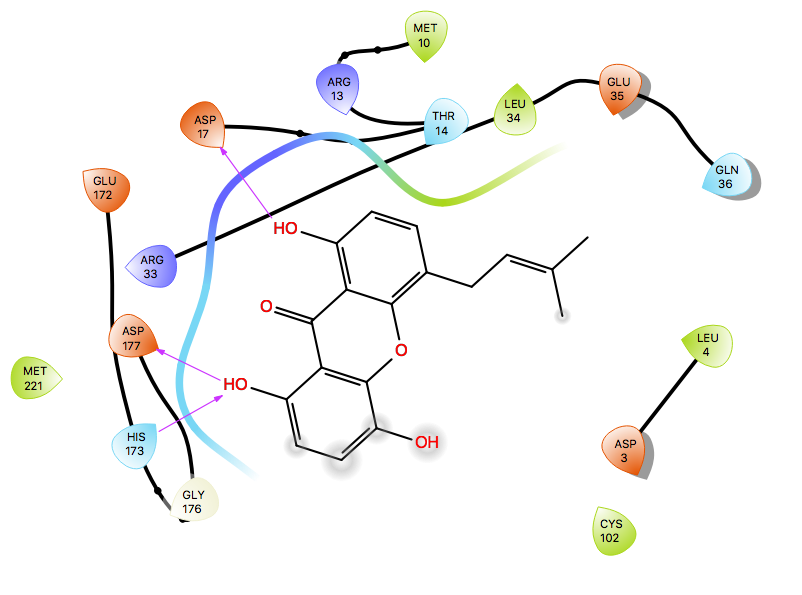
A B


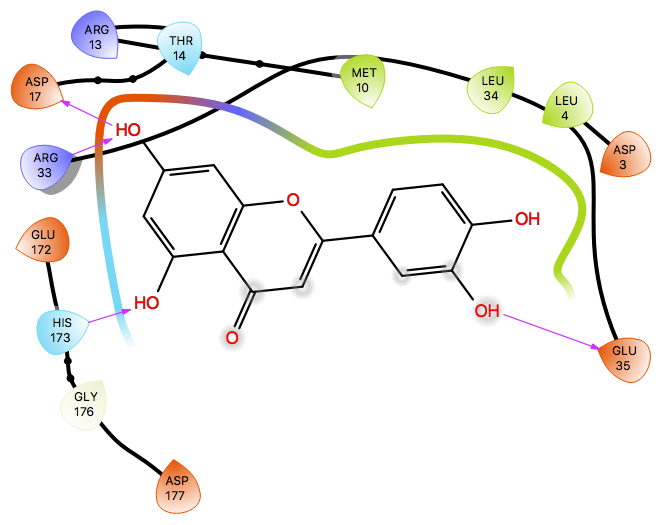

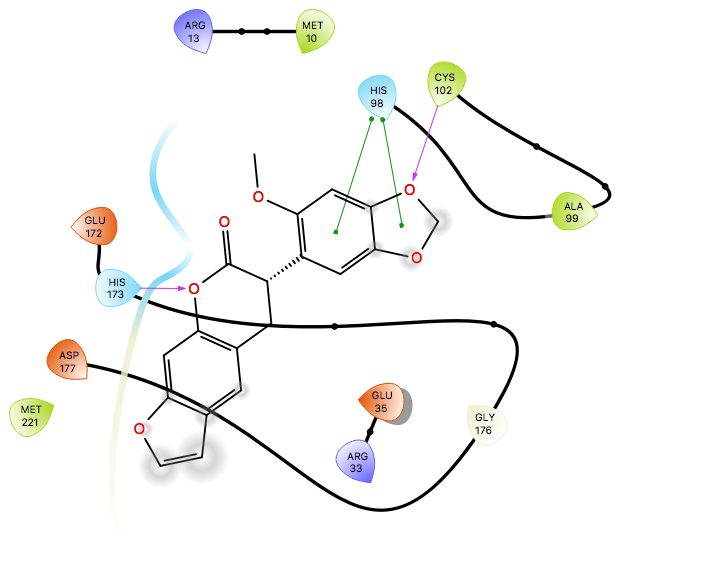
C D

Figure S4: Interaction maps (I – map) of the poses of the ligand complexes of the lead compounds docked in the metal binding site 2 of the modeled ideR for M. *ulcerans* obtained after IFD*.* The I – map of (A) ZINC000095485921, (B) ZINC000014417338, (C) ZINC000005357841 and (D) ZINC000018185774. Ligands are represented in gray.
